# Draft Genome Sequence of *Curtobacterium* sp. Strain UCD-KPL2560 (Phylum *Actinobacteria*)

**DOI:** 10.1128/genomeA.01040-16

**Published:** 2016-10-06

**Authors:** Brian A. Klein, Katherine P. Lemon, Lina L. Faller, Guillaume Jospin, Jonathan A. Eisen, David A. Coil

**Affiliations:** a Department of Microbiology, The Forsyth Institute, Cambridge, Massachusetts, USA; b Division of Infectious Diseases, Boston Children’s Hospital, Harvard Medical School, Boston, Massachusetts, USA; c Genome Center, University of California Davis, Davis, California, USA; d Department of Evolution and Ecology and Department of Medical Microbiology and Immunology, University of California Davis, Davis, California, USA

## Abstract

Here, we present the draft genome sequence of the actinobacterium *Curtobacterium* sp. strain UCD-KPL2560, which was isolated from the running surface of an indoor track field house in Medford, MA, USA (42.409716°N, -71.115169°W). The genome assembly contains 3,480,487 bp in 156 contigs.

## GENOME ANNOUNCEMENT

Members of the genus *Curtobacterium*, which was accepted as a genus in 1972 and refined in 1986 (1, 2), have been previously isolated from soil (3, 4), plants (5), cheese-processing equipment (6), and residential carpet (7). *Curtobacterium* spp. are characterized as Gram-positive aerobic bacilli that frequently demonstrate yellow-orange pigment when grown *in vitro* (2). *Curtobacterium* spp. are not common pathogens of humans; however, *Curtobacterium* spp. are recognized plant pathogens (8), and rare plant-to-human infections, as well as occasional human host isolation, are reported (9, 10).

We isolated *Curtobacterium* sp. strain UCD-KPL2560 from the flooring of an indoor track facility in Medford, MA, on 15 October 2015 as part of a project to produce reference genomes for microorganisms residing in the built environment. A nylon-flocked swab (Copan) dipped in sterile buffer (0.1 M NaCl and 0.1% Tween) was rubbed over the track surface starting block area (5 cm^2^) for 1 min, inoculated onto brain heart infusion agar containing fosfomycin (20 µg/ml), and incubated aerobically at 37°C for five days. A small round orange colony was selected from the original isolation plate and subcultured for purity. For 16S rRNA identification, an initial DNA extraction was performed with the DNeasy purification kit (Qiagen). A putative genus name was assigned to the isolate following PCR amplification (primers 27F and 1492R) and subsequent Sanger sequencing of the 16S rRNA gene. Genomic DNA for whole-genome sequencing was extracted using the MasterPure complete DNA/RNA purification kit (Epicentre).

Illumina paired-end libraries were generated using a Nextera DNA sample prep kit (Illumina). We selected 600- to 900-bp fragments using a Pippin Prep (Sage Science). Resultant libraries were sequenced on an Illumina MiSeq, with a read length of 300 bp. This produced a total of 3,920,338 paired-end reads. Quality trimming and error correction of the reads resulted in 3,506,299 high-quality reads using the A5-MiSeq assembly pipeline (version 05/22/2015) (11, 12). The resulting assembly contained 156 scaffolds (minimum, 598 bp; maximum, 215,854 bp; *N*_50_, 36,322 bp). The final assembly contained 3,480,487 bp, with a G+C content of 72% and a coverage estimate of 114% (EC value). Genome completeness was assessed using PhyloSift and CheckM (13, 14); all 37 PhyloSift marker genes were present, with 36 in single copy, and the ribosomal subunit L14 present in four copies. CheckM gave a 99% completeness estimation.

Annotation was performed using the RAST server (default settings on 14 April 2016) (15). *Curtobacterium* sp. strain UCD-KPL2560 contains 3,201 predicted coding sequences (CDSs), four predicted rRNAS, and 53 predicted tRNAs. It has 12 type IV pilus genes, 34 motility genes, and four auxin biosynthesis genes. At least two partial phages are predicted, and no clustered regularly interspaced short palindromic repeat (CRISPR) systems were identified (15–17).

We attempted to assign a putative species designation to *Curtobacterium* sp. UCD-KPL2560 using PhyloPhlAn and by generating a 16S-rRNA-gene-based phylogeny of *Curtobacterium* species curated by the Ribosomal Database Project (18, 19). However, neither yielded an assignment. PhyloPhlAn generated “*Clavibacter michiganensis*” taxonomic assignments for 16 of 17 *Curtobacterium* spp., with available NCBI sequencing data (one reclassified as *Rothia*). Additionally, *Curtobacterium* sp. UCD-KPL2560 did not fall within a well-supported clade of a single species within the genus *Curtobacterium* using 16S rRNA alone.

### Accession number(s).

This whole-genome shotgun project has been deposited at DDBJ/ENA/GenBank under the accession no. MCIG00000000. The version described in this paper is version MCIG01000000.
